# Solid Tumor-Targeted Infiltrating Cytotoxic T Lymphocytes Retained by a Superantigen Fusion Protein

**DOI:** 10.1371/journal.pone.0016642

**Published:** 2011-02-02

**Authors:** Jialin Sun, Lina Zhao, Lijie Teng, Feng Lin, Hongna Zhang, Zhengnan Li, Qiang Gao

**Affiliations:** Key Laboratory of Industrial Microbiology, Ministry of Education, College of Biotechnology, Tianjin University of Science and Technology, Tianjin, People's Republic of China; University of Rochester, United States of America

## Abstract

Successful immune-mediated regression of solid tumors is difficult because of the small number of cytotoxic T lymphocytes (CTLs) that were traffic to the tumor site. Here, the targeting of tumor-specific infiltrating CTLs was dependent on a fusion protein consisting of human epidermal growth factor (EGF) and staphylococcal enterotoxin A (SEA) with the D227A mutation. EGF-SEA strongly restrained the growth of murine solid sarcoma 180 (S180) tumors (control versus EGF-SEA, mean tumor weight: 1.013 versus 0.197 g, difference  = 0.816 g). In mice treated with EGF-SEA, CD4^+^, CD8^+^ and SEA-reactive T lymphocytes were enriched around the EGFR expressing tumor cells. The EGF receptors were potentially phosphorylated by EGF-SEA stimulation and the fusion protein promoted T cells to release the tumoricidal cytokines interferon-γ (IFN-γ) and tumor necrosis factor-α (TNF-α). Intratumoral CTLs secreted cytolytic pore-forming perforins and granzyme B proteins near the surface of carcinomas, causing the death of many tumor cells. We additionally show that labeled EGF-SEA was directly targeted to the tumor tissue after intravenous (i.v.) injection. The findings demonstrate that antibody-like EGF-SEA plays an important role in arresting CTLs in the solid tumor site and has therapeutic potential as a tumor-targeting agent.

## Introduction

The major lymphocytes in the blood are T cells. Usually, the T cell receptors (TCRs) of T cells cannot recognize self antigens on the surface of cancer cells. Thus, T cells moving through solid tumors in blood vessels are not reactive to cancer-associated antigens. The induction of tumor-reactive T cells has been attempted by various strategies, such as the use of CD3-based bispecific antibodies [1]–[3], and antibodies conjugated with a superantigen such staphylococcal enterotoxin A (SEA) [4]–[6]. However, the question of how to recruit large pools of effector T lymphocytes to solid tumors in order to facilitate tumor infiltration is still an attractive challenge. The mechanisms underlying tumor eradication that depend on the infiltration of cytotoxic T lymphocytes (CTLs) to solid tumors remain largely unknown. The movement of antibodies after intravenous (i.v.) injection has not been clearly shown. Thus, it is an open question as to whether only the tumor is targeted by the antibodies or whether other organs would retain them as well.

We developed a novel antibody-like fusion protein composed of human epidermal growth factor (EGF) and staphylococcal enterotoxin A (SEA) and examined its targeting to tumors and the tumoricidal immunostimulatory responses. The EGF receptor (EGFR) is an attractive target for therapy because EGFRs are often overexpressed in carcinomas [7]. Thus a cytotoxic agent that binds this receptor may have therapeutic applications. EGF and diphtheria toxin fusion proteins have been evaluated for their ability to induce regressions of subcutaneous human glioblastomas in athymic nude mice [8]. Additionally, EGF hybrids carrying pseudomonas exotoxin or ribonuclease displayed selective cytotoxicity against tumor cells bearing elevated EGFRs [9], [10]. These chimeric EGF proteins functioned as tumor-targeting molecules, but they were not dependent on antitumor immunocytotoxicity. SEA can activate a large number of T cells carrying a particular TCR variable β chain [11] and induce a strong cytokine production and CTLs in the CD4^+^ and CD8^+^ groups [4], [5].

The SEA gene employed in this study carried the D227A mutation. This mutant created by Dohlsten's group displayed a 1,000-fold reduction in major histocompatibility complex class (MHC) II binding to reduce systemic toxicity [6] and was consequently conjugated with antibodies as a powerful CTL inducer against cancer [6], [12], [13]. These fusion protein or antibodies fused with unmutant SEA [4], [5], [14], [15] inhibited blood carcinomas and metastases. Here by using fusion with EGF to target SEA (D227A) to the solid tumor, it becomes possible to retain tumor-unspecific T lymphocytes in the tumor site.

## Materials and Methods

### Protein preparation

A synthetic DNA fragment (Takara, Dalian, China) encoding a short linker peptide VDKLGGGGSGGGGSGGGGS, and the SEA gene encoding 233 amino acids [16] with the D227A mutation [6] were integrated into a modified pET22b (containing E-tag sequence GAPVPYPDPLEPR between the *Not* I and *Xho* I sites from pCANTAB 5E of the antibody phage display system, GE Healthcare Life Sciences, Piscataway, NJ, USA) at the *Hin*d III and *Not* I sites to produce SEA. Another synthetic human EGF DNA fragment (Takara, Dalian, China) encoding 53 amino acids [17] was inserted at the *Eco*R I and *Hin*d III sites of pET22b-SEA to produce the fusion protein EGF-SEA. The molecular weights of SEA and EGF-SEA including the leader and upstream sequences in pET22b were estimated as 34.6 and 41.2 kDa, respectively. Two proteins, which expressed as inclusion bodies in *Escherichia coli* L21(DE3) strain, were purified using immobilized metal ion (Ni^2+^) affinity chromatography, refolded using to the method of GSSG and arginine dialysis [18], and resolved by 10% sodium dodecyl sulfate-polyacrylamide gel electrophoresis (SDS-PAGE).

### Murine tumor model

Male ICR mice (Experimental Animal Center, Academy of Military Medical Sciences, Beijing, China), 4–5 weeks old and 18–22 g, were employed in the tumor model. All mice were maintained in a specific pathogen free (SPF) facility at Tianjin University of Science & Technology. The animal experiments were approved by the Institutional Biosafety Committee and the Animal Care according to the guideline on animal research (No. 503012) and Use Committee of Tianjin University of Science & Technology with a permit number of TUST070824. Mouse sarcoma 180 (S180) cells were from the American Type Culture Collection (Manassas, VA). Each mouse was subcutaneously inoculated with 2×10^6^ S180 cells suspended in phosphate-buffered saline (PBS) into the right axilla. Mice were divided into three groups (n = 15 per group) and given two daily intraperitoneal (i.p.) injections (0.2 ml) with 0.9% NaCl saline (control), SEA (250 pmol) and EGF-SEA (250 pmol), starting on day 2 after tumor inoculation. The tumors were measured on day 9.

### Fluorescent labeling of EGF-SEA

Purified EGF-SEA proteins were labeled with Kodak X-SIGHT 670 TFP ester (Carestream Health, Rochester, NY, USA) according to the protocol (Carestream Molecular Imaging). Briefly, dyes were conjugated with proteins in a sodium phosphate buffer (100 mM, 0.15 M NaCl, pH 7.2) and unconjugated dyes were neutralized by ammonium chloride. The labeled proteins were purified from free dyes on a Sephadex G-25 column. The ratio of free dyes to the proteins (degree of labeling) was estimated by measuring absorbance at 280 nm and 670 nm.

### Flow cytometry

S180 cells were collected, centrifuged at 1,500 rpm, and re-suspended in PBS. Aliquots of 2×10^6^ cells were incubated with 125.00, 12.50 and 1.25 pmol of LSS670-labeled EGF-SEA proteins, respectively, at 4°C for 30 min, and washed in cold PBS. The ability of labeled-EGF-SEA to bind to S180 cells was quantified by fluorescence-activated cell sorter (FACS) analyses using a BD FACS Calibur (Becton Dickinson Medical Devices, Franklin Lakes, NJ, USA).

### Immunohistochemistry

Tissues from 8 mice per group were fixed in 4% phosphate-buffered formalin, embedded in paraffin, sectioned at 5 µm, deparaffinized in xylene and then rehydrated in graded ethanol using a general method. Sections were incubated first in a 3% hydrogen peroxide solution to block endogenous peroxidases, then with a protein-blocking solution containing preimmune rabbit serum, and finally with the following primary antibodies (1∶250 dilution in PBS containing 1% bovine serum albumin) in each test: rabbit CD4-specific polyclonal antibody, rabbit CD8-specific polyclonal antibody, rabbit interferon-γ (IFN-γ)-specific polyclonal antibody, rabbit tumor necrosis factor-α (TNF-α)-specific polyclonal antibody, rabbit Fas-specific polyclonal antibody, and rabbit perforin-specific polyclonal antibody from Santa Cruz Biotechnology (Cruz, CA, USA); mouse phospho-EGFR-specific monoclonal antibody (Y 1068) from Cell Signaling Technology (Danvers, MA, USA); rabbit granzyme B-specific polyclonal antibody from Abcam (Cambridge, MA, USA); goat anti-mouse or anti-rabbit secondary antibody from Invitrogen (Carlsbad, CA, USA). To prepare SEA-specific antibodies, SEA purified from the recombinant *E. coli* was raised in BALB/c mice, and the IgG fraction from the antiserum with a titer over 8000 was purified using a Hitrap Protein G-Sepharose column (GE Healthcare Life Sciences, Piscataway, NJ, USA). Antibody-binding was detected by incubation with the secondary antibody for 1 hr, and then avidin-biotin-peroxidase complex (Zymed, San Francisco, CA, USA) for 30 min followed by diaminobenzidine (DAB) for 8 min. The slides were rinsed with PBS and counterstained with hematoxylin for 1 min. About 15–20 sections were analyzed for each antibody assay, and sections detected for T cells, cytokines, Fas and granulolytic proteins were from the same tumor.

### Cytokine release

Blood and spleen samples were collected after the mice were dissected. All groups contained pooled sera and spleens from at least three mice. Protein levels of TNF-α and IFN-γ were measured using specific enzyme-linked immunosorbent assay (ELISA) kits (R & D Systems, Minneapolis, MN, USA) according to instructions from the manufacturer.

### TUNEL staining

To assess fragmented DNA, sections were labeled by the terminal deoxynucleotidyl transferase biotin-dUTP nick-end labeling (TUNEL) technique using an In Situ Cell Death Detection Kit (Roche Applied Science, Mannheim, Germany). Additional sections were incubated with terminal deoxynucleotidyl transferase (TdT), a fluorescein-labeled nucleotide mixture, in a wet chamber at 37°C for 1 hr. The sections were then washed with PBS and incubated with anti-fluorescein antibody conjugated with horseradish peroxidase (POD) for 30 min.

### Imaging of mice

EGF-SEA proteins (125 pmol) labeled by LSS670 were injected i.v. via the tail vein into mice bearing tumors of 0.5∼1.0 cm in diameters or into mice that had not been inoculated with S180 cells. Mice were anesthetized and the targeting of labeled proteins to tumors was monitored for 62 hrs using *in vivo* fluorescence imaging IVIS Kinetic (Caliper Life Sciences, Hopkinton, MA, USA) with an excitation bandpass filter at 710 nm and collecting emissions from 810 to 885 nm.

## Results

### Inhibition of the growth of solid murine S180 tumors

**Figure 1:**
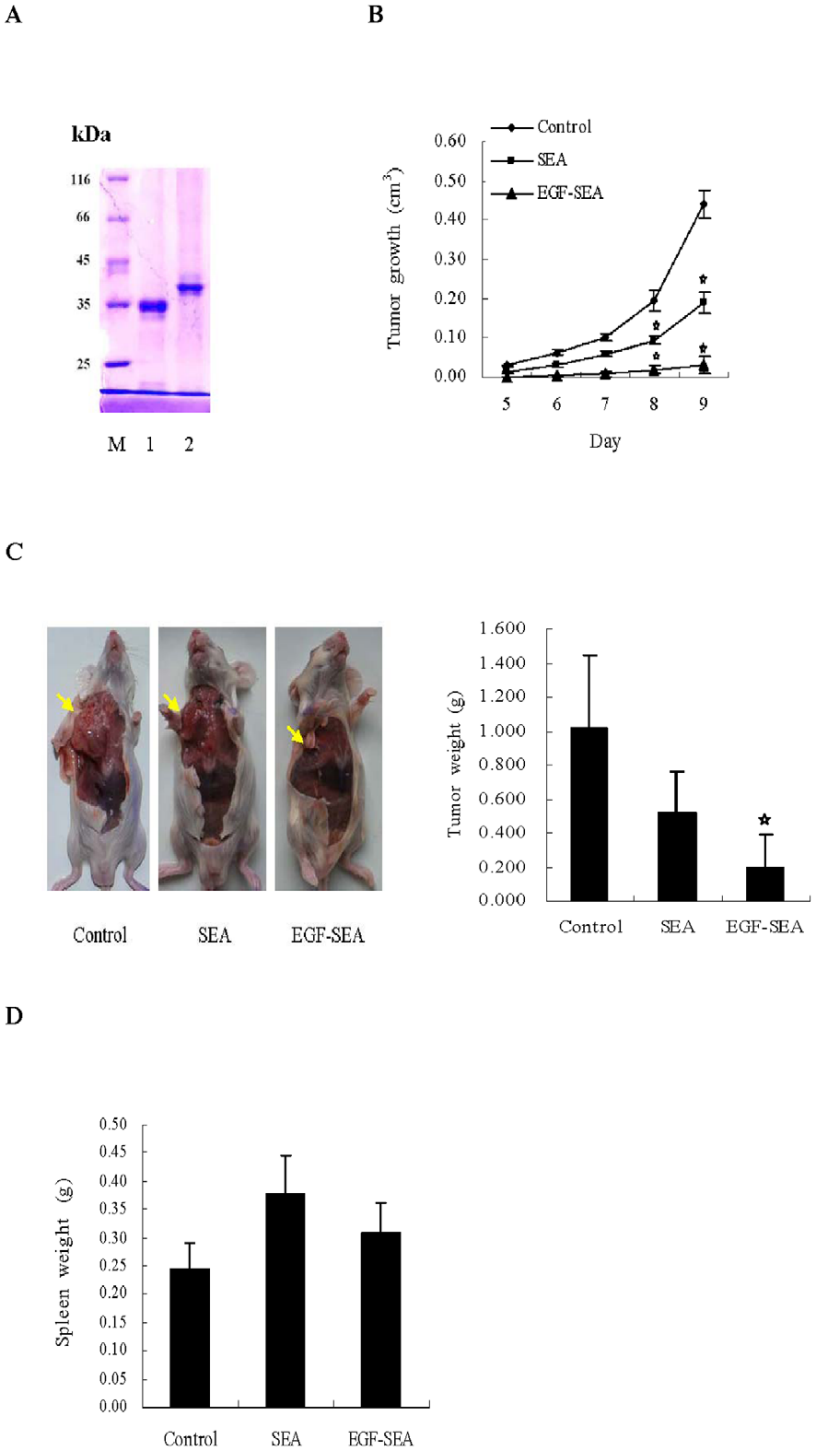
Inhibition of S180 tumor growth. (A) SDS-PAGE identification of proteins. Lane 1, SEA; lane 2, EGF-SEA. (B) Effect of treatments on the growth of S180 tumors. After mice were inoculated with 2×10^6^ S180 cells into the right axilla, they were treated with two daily i.p. injections (0.2 ml) of 0.9% NaCl saline (control), SEA (250 pmol) and EGF-SEA (250 pmol) starting 1 day after tumor inoculation. n = 15 mice per group. *p<0.001. (C) Inhibition of S180 tumors. Mice were dissected and the tumors were measured on day 9. Yellow arrows indicated the tumor position. *p<0.001. (D) Effect of treatments on spleens.

Human EGF and SEA gene fragments were subcloned into pET22b and expressed in *E. coli*. EGF-SEA and SEA products were then purified and refolded. The proteins secreted into the periplasmic space of *E. coli* were not detected. The molecular weights of SEA and EGF-SEA were close to 35 kDa and over 40 kDa, respectively (Figure 1A) and the trace of the proteins below two major bands of the proteins may be cleavaged by a signal peptidase, whereas the major proteins formed as inclusion bodies may be not processed. The refolded proteins were utilized in animal tumor model.

To examine antitumor cytotoxicity induced by EGF-SEA, S180 cells were subcutaneously inoculated into the right axilla of ICR mice, followed by i.p. injections of saline (control), SEA, or EGF-SEA, respectively. The solid tumors appeared on day 5 and quickly expanded over the next 2 days in the control mice, while the growth of tumors was delayed in mice treated with EGF-SEA (Figure 1B). The tumors in mice treated with SEA developed with a 1–2 day delay compared to the control mice. EGF-SEA strongly suppressed solid tumor growth (control versus EGF-SEA, mean tumor weight: 1.013 versus 0.197 g, difference  = 0.816 g, 95% confidence interval [CI]  = 0.54 to 1.37, p<0.001) (Figure 1C), and had a slight influence on the spleen measured by weight (Figure 1D). The control/EGF-SEA tumor weight ratio was 5.14.

### Interaction between EGF-SEA proteins and S180 cells

**Figure 2:**
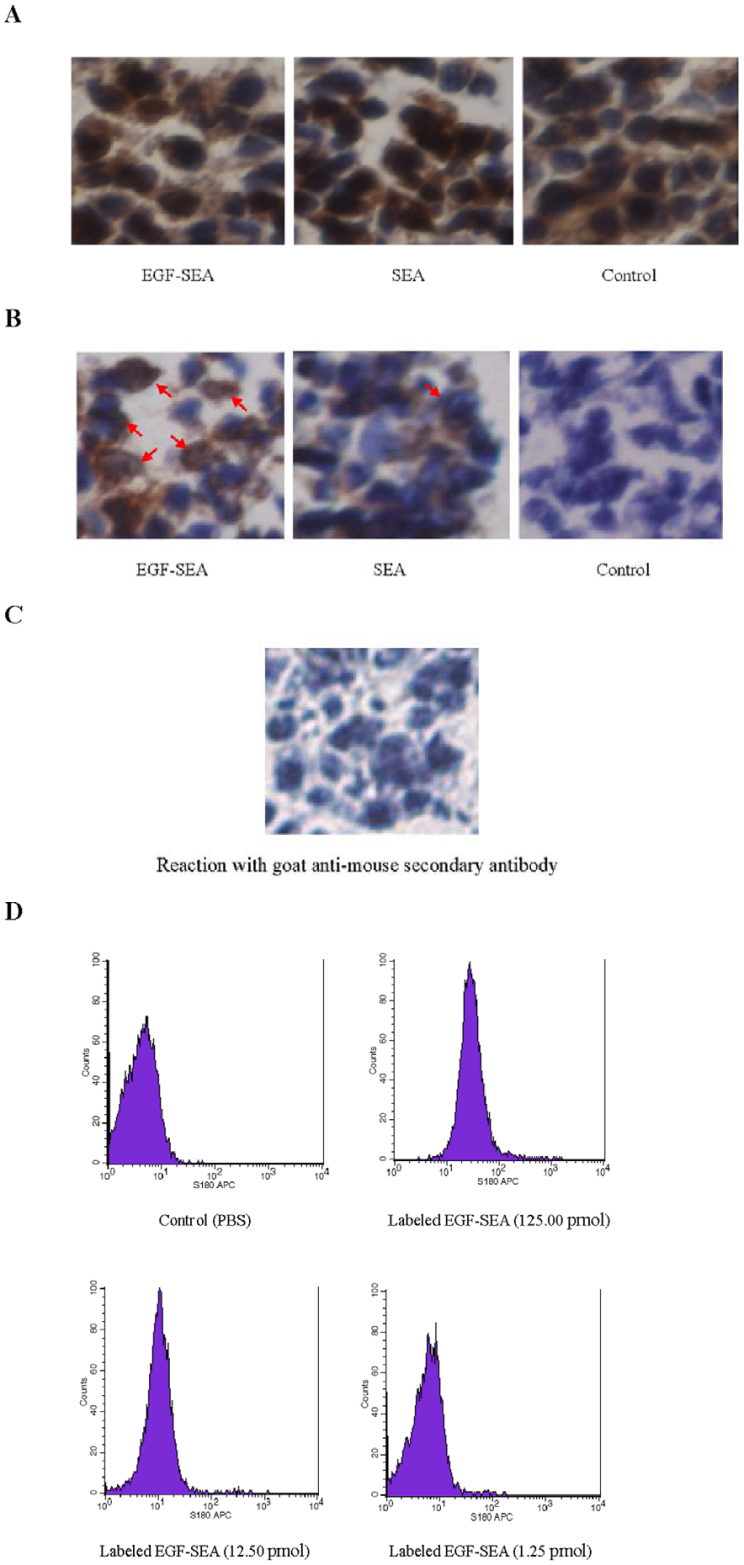
Interactions between EGF-SEA and EGFRs expressed by S180 carcinomas. (A) EGF-reactive S180 tumor cells and SEA-reactive T cells. Sections were incubated with an EGF-SEA solution, followed by mouse polyclonal anti-SEA IgG fraction and anti-mouse secondary antibody. Anti-SEA antibodies were able to react with EGF-SEA on the surface of S180 tumor cells and T lymphocytes (small brown spots in the section treated with EGF-SEA). (B) Detection of phospho-EGFR (Y 1068) in S180 cells. Sections were labeled with mouse phospho-EGFR-specific monoclonal antibody (Y 1068). Phosphorylated EGFR molecules were found in the tumors treated with EGF-SEA and SEA (red arrows). (C) An immunohistochemistry control. A tumor section from the EGF-SEA treated group was labeled with goat anti-mouse secondary antibody only. (D) Analysis of LSS670-labeled EGF-SEA binding to S180 cells by FACS analysis. S180 cells (2×10^6^) were incubated with 125.00, 12.50 and 1.25 pmol of LSS670-labeled EGF-SEA proteins and PBS (control). Microscope magnification: ×400 in A to C.

Immunohistochemistry performed after incubation of tumor sections with an EGF-SEA solution and detection by anti-SEA IgG demonstrated that large tumor cells and T cells (small spots) in tumors were associated with EGF-SEA proteins (Figure 2A), whereas no positive cells were detected in a section treated only with the anti-mouse secondary antibody (Figure 2C). Because of EGF-SEA containing an E-tag peptide, positive tumor cells and T cells were also found using anti-E-tag IgG detection (data not shown).

The phosphorylation of EGFR at Tyr-1068 occurred in many S180 cells of the EGF-SEA treated group, but was low and negligible in the SEA and control groups, respectively (Figure 2B). In a control of the immunohistochemistry, no labeling was observed in a tumor treated with EGF-SEA using only the goat anti-mouse secondary antibody (Figure 2C).

The ability of EGF-SEA to bind to S180 cells was directly demonstrated using FACS analysis. Nearly 95% of the S180 cells (2×10^6^) were coated with LSS670-labeled EGF-SEA molecules after incubation with 125 pmol of EGF-SEA proteins (Figure 2D). S180 cells bound labeled EGF-SEA proteins decreased with reduction in the protein concentration.

### CD4^+^, CD8^+^ and SEA-reactive T cells and cytokine secretions

**Figure 3:**
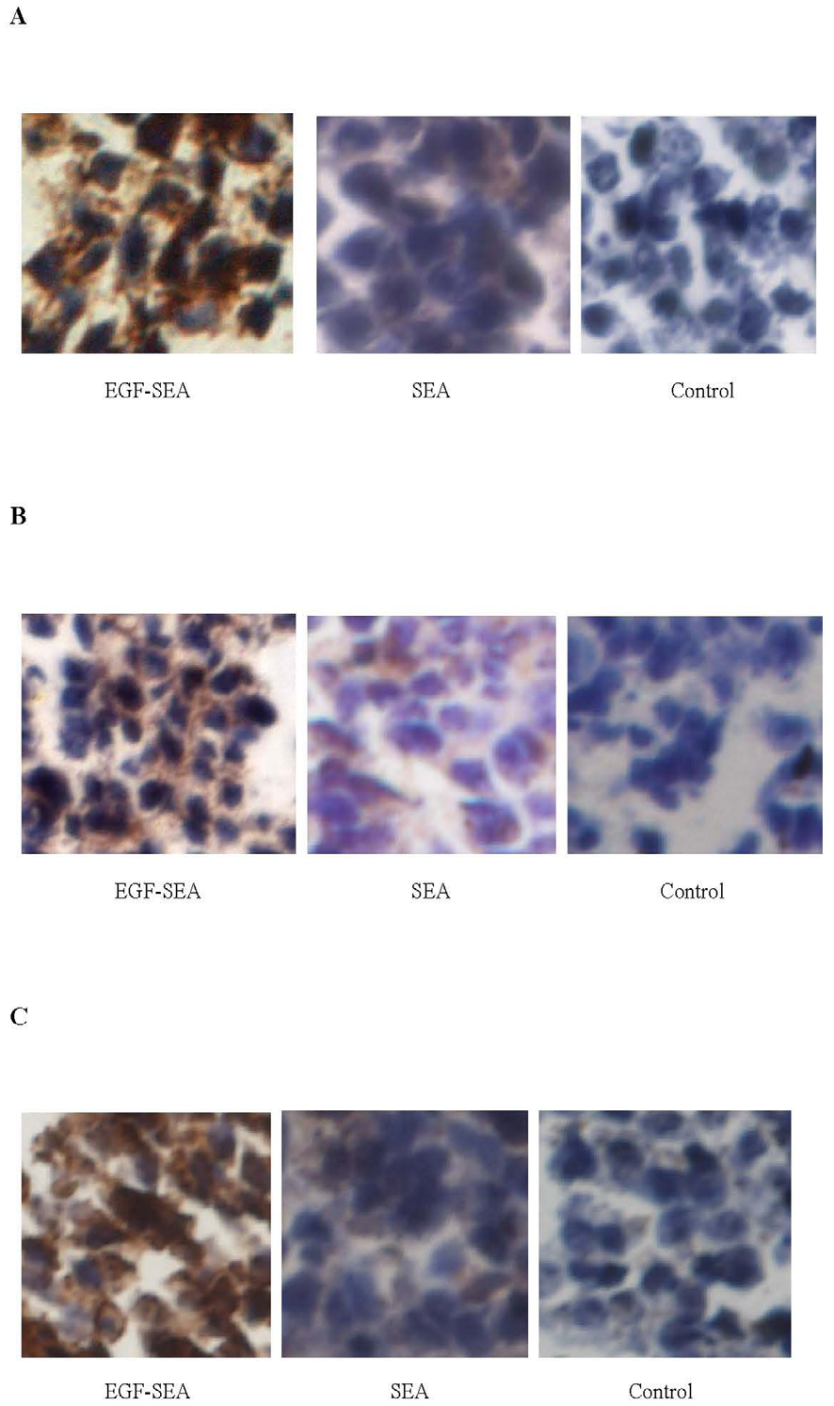
Detection of intratumoral CTLs. Small brown spots around S180 tumor cells indicated antibody-reactive T cells. (A) CD4-positive cells detected by rabbit CD4-specific polyclonal antibody. (B) CD8 positive cells detected by rabbit CD8-specific polyclonal antibody. (C) SEA-reactive T cells. Sections were incubated with a SEA solution, followed by mouse polyclonal anti-SEA IgG fraction and anti-mouse secondary antibody. Microscope magnification: ×400 in A to C.

Infiltrating T lymphocytes were detected in S180 tumors treated with EGF-SEA and found to be CD4^+^, CD8^+^ and SEA-reactive (Figure 3). Large positive spots judged to be S180 cells reflected that abundant T cells were presented on the surface of tumor cells. The number of SEA-reactive T cells seemed to be close to or over the sum of CD4^+^ and CD8^+^ T cells. Only few and negligible T cells were detected in the tumors of SEA and control groups, respectively.

**Figure 4:**
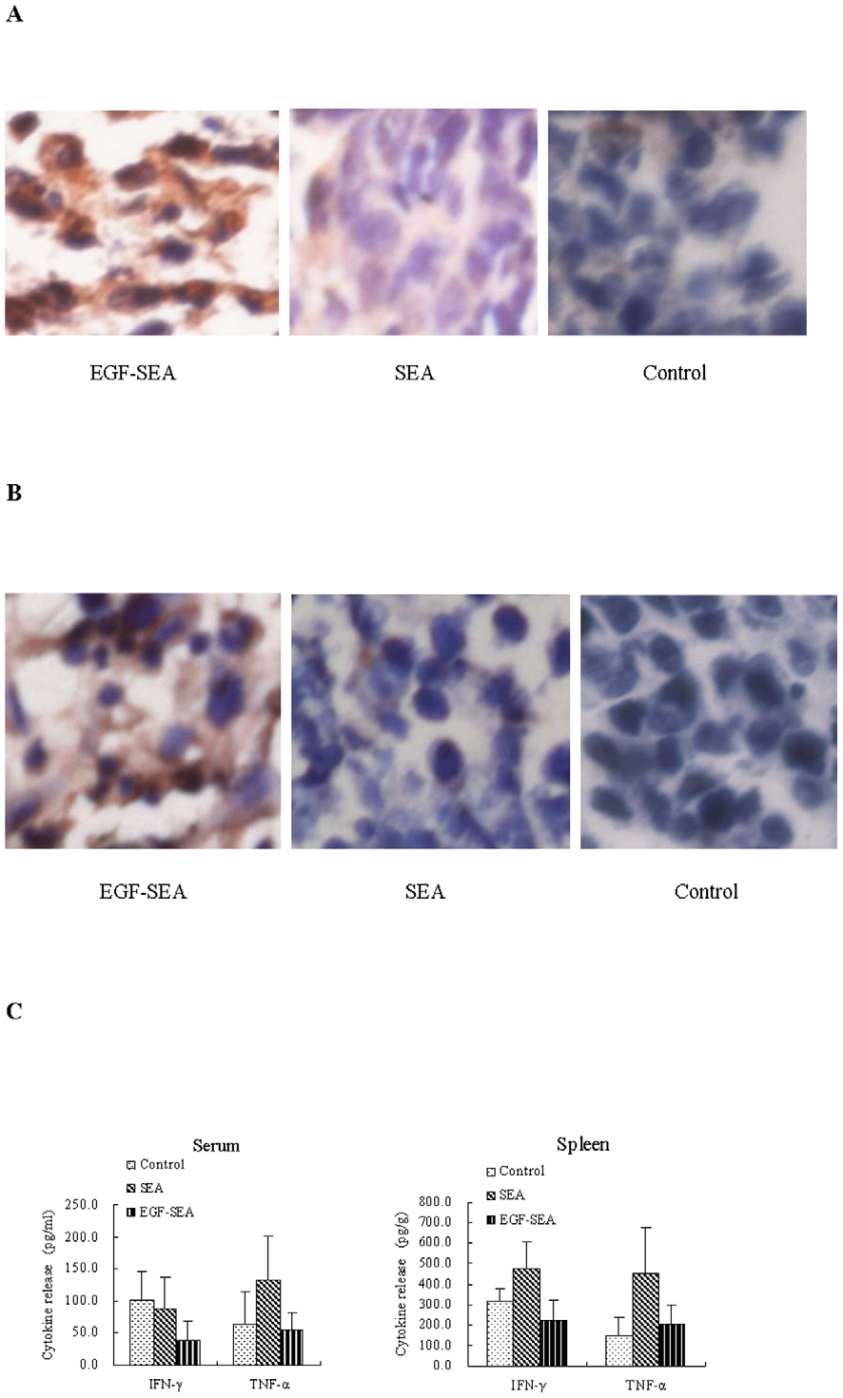
Cytokine release in mice bearing S180 tumors. (A) TNF-α secretion in tumors. The sections were labeled with rabbit TNF-α-specific polyclonal antibody. (B) IFN-γ secretion in tumors. The sections were labeled with rabbit IFN-γ-specific polyclonal antibody. (C) Cytokine secretion in mice. The data represented cytokine levels in sera in pg/ml and in spleens in pg/g. Cytokines in blood and spleen samples were measured using specific ELISA kits. Microscope magnification: ×400 in A and B.

In tumors treated with EGF-SEA, there was a large increase in secretion of TNF-α and IFN-γ around the S180 cells (Figure 4A and B), whereas the levels of the blood and spleen were relatively low (Figure 4C). In contrast, high levels of cytokines were found in blood and spleen of mice treated with SEA.

### Apoptosis of tumor cells

**Figure 5:**
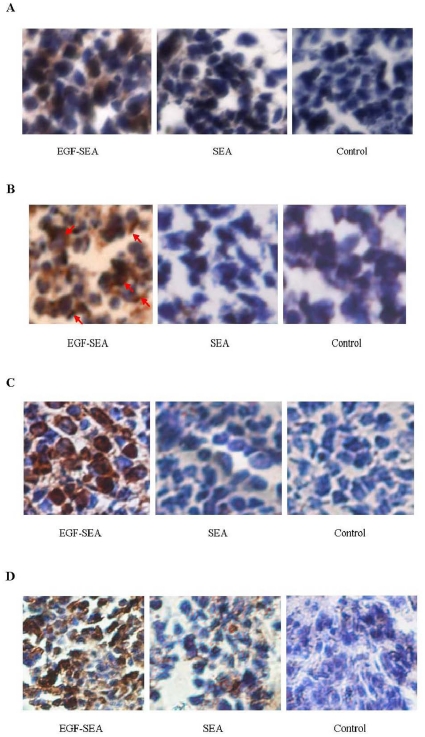
Apoptosis of S180 carcinomas. (A) Detection of Fas expression in S180 tumor cells using rabbit Fas-specific polyclonal antibody. Fas molecules were detected on the surface of S180 cells (brown staining). (B) Perforin secretion by T cells in S180 tumors. Perforins were detected by rabbit perforin-specific polyclonal antibody, and found to be pore-forming (red arrows). (C) Secretion of granzyme B by T cells in S180 tumors. Granzyme B proteins were detected by rabbit granzyme B-specific polyclonal antibody. (D) Cell death in S180 carcinomas. Apoptotic tumor cells were detected by TUNEL staining. Microscope magnification: ×400 in A to D.

In addition to this local increase in cytokine secretion in treated tumor, immunohistochemistry analysis showed that Fas expression was upregulated and largely restricted in the tumors treated with EGF-SEA (Figure 5A). Activated T cells secreted pore-forming perforins onto the target and lytic granzyme B granules concentrated on the membrane of S180 cells (Figure 5B and C). As according to a perforin report [19], the molecules assembled a strip were judged to be perforin. Through the direct killing mediated by perforins and granzyme granules acting together with the apoptotic inducers, TNF-α and Fas (Figure 5A), tumor cells were eliminated in EGF-SEA-treated mice, as demonstrated by TUNEL staining (Figure 5D).

### *In vivo* imaging of EGF-SEA targeting to solid tumors

**Figure 6:**
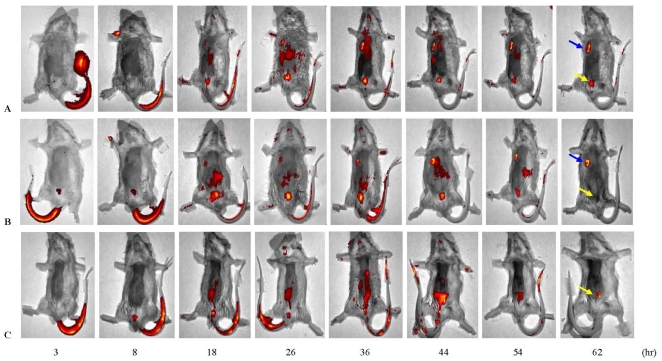
Monitoring of the targeting of EGF-SEA to solid tumors by *in vivo* imaging of mice. LSS670-labeled EGF-SEA proteins (125 pmol) were injected i.v. into mice bearing tumors (A and B) and un-inoculated mice as control (C). Movement of labeled EGF-SEA proteins was observed using *in vivo* fluorescence imaging with the IVIS Kinetic imaging system. Blue and yellow arrows indicated the tumor and bladder positions.

To avoid dye-labeling background and show a clear route of EGF-SEA movement in mice, LSS670-labeled EGF-SEA was injected i.v. via the tail vein. In the initial 8 hrs after the injection, the protein stayed in the tails of mice (Figure 6). The protein then diffused to the abdomen and bosom over a course of 18 to 54 hrs, reaching the tumors 18 hrs after the injection. The bladder stored excretive proteins with the peak retention occurring from 26 to 36 hrs and then gradually decreasing after 44 hrs. Finally, abundant proteins accumulated in the tumor tissue, but there was negligible deposition in other organs except bladder containing the remaining traces of the unexcreted proteins. In a mouse that had not been inoculated with S180 carcinomas, labeled protein was largely removed via bladder from the body, although there was random diffusion of the protein.

## Discussion

Enhanced EGFR expression has been found in a wide variety of malignancies, including esophageal, lung, thyroid, gastric, bladder, renal, prostate, ovarian, endometrial and breast. One mucinous adenocarcinoma was shown to have an extreme elevation in levels of EGF receptors, corresponding to a 320-fold increase over normal mucosa [7]. The amount of EGFR expressed on S180 cells is likely to be abundant as a large amount of the EGF-SEA protein accumulated in the tumor site, peaking 62 hrs after i.v. injection (Figure 6). In contrast, the protein did not persist in other organs except the bladder containing traces of excreted or degraded EGF-SEA proteins, indicating that only the tumor had highly affinity for EGF-SEA and acted as a reservoir in which EGF-SEA molecules flowing through blood vessels were stored.

Although there are cross-interactions between EGF family and their receptors from human and mouse based on the structural similarity [17], [20], such as human EGF binding the mouse EGF receptor [21] and mouse EGF binding the human EGF receptor [22], we confirmed that the human EGF peptide of the EGF-SEA fusion protein bound the EGFR of S180 cells by showing the interaction of anti-SEA antibody with EGFR-interacting EGF-SEA molecules (Figure 2A) and performing FACS analysis (Figure 2D). Moreover, labeling with a phospho-specific antibody showed that EGF-SEA probably caused EGFR phosphorylation at Tyr-1068 (Figure 2B).

The first injection of EGF-SEA solution was performed 2 days after inoculation of mice with S180 cells and the EGF-SEA protein reached the colonial S180 carcinomas 18 hrs later (Figure 6). Thus, there were 18 hrs for S180 cells to develop into a growing tumor before the arrival of EGF-SEA. Once the tumor formed a site for the retention of EGF-SEA proteins, it was possible for tumor-unspecific T lymphocytes including CD4^+^ and CD8^+^ T cells to be likewise retained. More carcinoma-associated EGF-SEA molecules resulted in more captured T cells neighboring to or anchored on the surface of S180 tumor cells. After accumulation, the T cells stimulated by EGF-SEA became tumor-sensitive CTLs that secreted the tumoricidal cytokines TNF-α and IFN-γ (Figure 4), and the granulolytic proteins perforins and granzyme B (Figure 5). Additionally, tumor cells were found to have increased expression of Fas, which might be up-regulated by IFN-γ and TNF-α [23]–[25]. Perforin is a granule protein capable of forming transmembrane pores that are able to play as passive directors of granzymes into the target cell membrane and also allow an ionic exchange that causes osmotic imbalance and in consequence, cell death [26], [27]. Granzymes are released as a multi-molecular complex, inducing apoptosis by caspase-independent or –dependent pathways [28], [29].

Increasing the intratumoral concentration of cytokines such as IFN-γ and TNF-α [30], [31] would directly enhance the cytotoxic immune responses against tumors while causing only limited systemic toxicity. In contrast, treatment with a non-specific SEA would irritate T lymphocytes in the whole body. In SEA-injected controls, some percentage of the T cells recirculated to the spleen, as shown by their distinct incidence on the spleen (Figure 1D) and increased cytokine levels in the spleen and blood (Figure 4C). There was also lower cytokine production in the tumors, as reflected by the detection of fewer T cells (Figure 3). Cytokine secretion seemed to rely upon the location where superantigen-activated T lymphocytes adhered and proliferated, demonstrating that the targeting of tumor-infiltrating CTLs by EGF-SEA was required for the restriction of cytokine cytotoxicity to the tumor site.

A question remains as to whether localization of lymphocytes to a tumor requires specific recognition of a tumor antigen in the vascular compartment or whether the initial recruitment is dependent on the characteristics of the tumor-associated vasculature. The importance of lymphocyte specificity during the initial recruitment of antitumor effector cells remains unclear. However, the ability of the leukocyte population to identify tumor-associated antigens and kill the cells that express them is not sufficient to affect a complete antitumor response in animal models. A possible explanation for this could be deficient homing of cells with necessary specificity to the tumor site [32].

Targeting CTLs to the site of tumors in animal models is a labor-intensive process. However, it is of great potential therapeutic interest because the frequency of tumor-specific effector T lymphocytes is generally insufficient to arrest progressive tumor growth. The majority of T cells are trapped in the liver, spleen or lungs, generating in low assembly of tumor-sensitive T lymphocytes in the tumor tissue [32]–[35].

In mice treated with saline or SEA, T lymphocytes without the specificity for tumor-associated antigens did not stop moving or eliminate tumor cells (Figure 5D), resulting in a lack of adequately intratumoral CTLs (Figure 3). The success of immunotherapeutic strategies against cancer depends on the generation of effective tumor-specific T lymphocytes that must not only enter the tumor area but also can traverse the interstitial region and directly interact with the target cells [36].

In the present work, S180 cells were inoculated into the right axilla of mice to induce solid tumor formation. After one day of colonization, EGF-SEA was administered by i.p. injections. Following a period of random diffusion, EGF-SEA molecules selectively concentrated in developing tumors that expressed sufficient EGFR, and then retained the passage of T lymphocytes within the tumor site. This retention of T cells effected their deep infiltration into solid tumors, even though the lymphocytes were unable to recognize tumor-autologous antigens specifically. Once EGF-SEA-promoting infiltrating CTLs accumulated in the tumor site, the tumor became apoptotic by an *in situ* attack. EGF-SEA thus established a bridge between CTLs and tumors, in which EGF targeted the fusion protein to tumors expressing abundant EGF receptors and SEA provoked the infiltrating CTLs into lysing solid tumors by granule exocytosis and a Fas-mediated death pathway. These findings suggest that close-contact accumulation of CTLs within the tumor site is required for effective tumor suppression.
